# Cloxacillin: A New Cause of Pill-Induced Esophagitis

**DOI:** 10.1155/2016/2904256

**Published:** 2016-04-14

**Authors:** Petros Zezos, Ziv Harel, Fred Saibil

**Affiliations:** ^1^Division of Gastroenterology, Department of Medicine, Sunnybrook Health Sciences Centre, University of Toronto, Toronto, ON, Canada; ^2^Division of Nephrology, Department of Medicine, St. Michael's Hospital, University of Toronto, Toronto, ON, Canada

## Abstract

A large variety of medications can cause pill-induced esophagitis. Herein we present a case of cloxacillin-induced esophagitis. A 66-year-old male presented with an acute onset of epigastric and retrosternal pain on the 5th day of a course of oral cloxacillin prescribed for erysipelas. Initial clinical and imaging assessment was negative and he was sent home. A few days later, he returned with persistent severe retrosternal pain; endoscopy at the same day revealed a normal upper esophagus, several small stellate erosions in the midesophagus, and a normal squamocolumnar junction with a small hiatus hernia. Treatment with esomeprazole 40 mg bid and Mucaine^R^ suspension resulted in complete resolution of his symptoms. Pill-induced esophagitis may be underreported by patients, when symptoms are mild and unrecognized and/or underdiagnosed by the clinicians as a cause of retrosternal pain, odynophagia, or dysphagia. Failure of early recognition may result in unnecessary diagnostic investigations and prolongation of the patient's discomfort. This case signifies the importance of enhancing clinician awareness for drug-associated esophageal injury when assessing patients with retrosternal pain, as well as the value of prophylaxis against this unpleasant condition by universally recommending drinking enough water in an upright position during ingestion of any oral medication.

## 1. Case Presentation

A 66-year-old male was referred to our department on an urgent basis with an acute onset of epigastric and retrosternal pain and loss of appetite. The pain began on the 5th day of a course of oral cloxacillin prescribed for the treatment of erysipelas. On the first day of pain, he presented to our emergency department (ED), where evaluation, including an abdominal CT scan, failed to reveal a cause, and he was discharged home. After 3 more days on cloxacillin, the patient suspected that the cloxacillin might be the cause of his symptoms and he discontinued it. He improved transiently, but 2 days later he awoke early in the morning with severe retrosternal pain, coming in waves. He returned to the ED and was referred to our service. He denied having prior episodes of food impaction or dysphagia. He did have a prior history of intermittent reflux symptoms managed with on-demand esomeprazole. He had several comorbidities, including hypertension, diabetes mellitus type II, and a recent diagnosis of a left pleural effusion of unknown cause which was resolving. His other medications included irbesartan, atorvastatin, and metformin. Endoscopy was performed the same day.

## 2. Procedure

**Figure 1:**
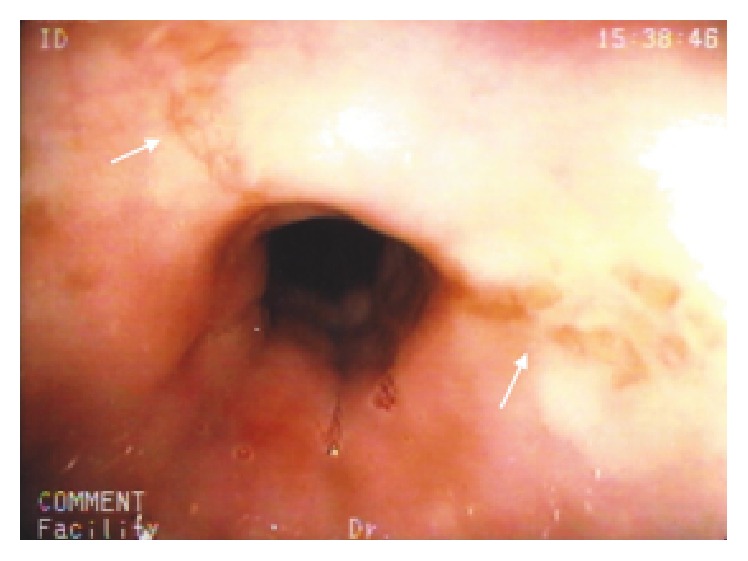
Stellate erosions in the midesophagus.

Upper gastrointestinal endoscopy revealed a normal upper esophagus, several small stellate erosions in the midesophagus (Figure 1), and a normal squamocolumnar junction with a small hiatus hernia. No biopsies were taken. We agreed with the patient's opinion that his symptoms were due to the cloxacillin and that the ulceration was likely causing esophageal spasm. We prescribed esomeprazole 40 mg bid and Mucaine^R^ suspension, a liquid antacid that contains a local anesthetic. We also prescribed nitroglycerine spray to be used just before each meal to try to prevent the episodes of spasm. We advised him to avoid ingesting anything very hot or very cold. A few days later, the patient called us and reported complete resolution of his symptoms.

## 3. Discussion

The literature is replete with cases of pill-induced esophagitis due to a large variety of medications. However, after a thorough literature search, we believe this is the first case report of cloxacillin-induced esophagitis.

Pill-induced esophagitis may be underreported by patients, when symptoms are mild and unrecognized and/or underdiagnosed by the clinicians as a cause of retrosternal pain, odynophagia, or dysphagia due to a lack of clinical awareness and suspicion. Failure of early recognition of this entity may result in unnecessary diagnostic investigations, as well as prolongation of the patient's discomfort. Delayed diagnosis may also result in more severe complications, including gastrointestinal bleeding, stenosis, or perforation. In most cases, the symptoms improve once the offending drug is recognized and withdrawn [1].

The diagnosis of pill-induced esophagitis is based on both clinical presentation and endoscopic findings. The most common presentation is acute onset retrosternal pain or heartburn, odynophagia, and dysphagia [1–3]. Theoretically, any retained pill to the esophagus can cause mucosal injury, but the most common offenders have been antibiotics, nonsteroidal anti-inflammatory drugs (NSAIDs), and antihypertensives [1–3]. Endoscopy typically reveals midesophageal ulcers [1–3], while the more frequent reflux-associated ulcers are located in the lower esophagus [4]. Aside from stopping the causative agent, the management can include short-term treatment with proton pump inhibitors, sucralfate, antacids, and avoidance of irritating food (very hot or cold and acidic). Prophylactic recommendations include swallowing drugs with enough water and avoiding the supine position when ingesting medications. Most patients respond to conservative therapy, and no other measures or follow-up endoscopy is necessary.

Our case is consistent with the presentation of pill-induced esophagitis.* Cloxacillin* is a broad-spectrum antibiotic belonging to the group of semisynthetic penicillins. Although cases of penicillin, ampicillin, and amoxicillin-induced esophagitis have all been reported [2, 3], there are no previous reports implicating cloxacillin. The gelatin capsule covering of many antibiotics, including cloxacillin, is sticky and thus prone to esophageal retention [3]. It is unclear if the mechanical pressure of the retained capsule against the esophageal wall or the contact and reaction of the drug released after the capsule dissolves leads to the mucosal injury [3]. Intravenous administration of cloxacillin causes endothelial damage and phlebitis [5], but the effect of the oral drug on the GI tract mucosa is unknown.

This case signifies the importance of enhancing clinician awareness for drug-associated esophageal injury when assessing patients with retrosternal pain, as well as the value of prophylaxis against this unpleasant condition by universally recommending drinking enough water, a minimum of 120 mL, during ingestion of any oral medication, and doing it in an upright position.
